# Direct Observation of the Epitaxial Growth of Bismuth Telluride Topological Insulators from One-Dimensional Heterostructured Nanowires

**DOI:** 10.3390/nano12132236

**Published:** 2022-06-29

**Authors:** Rei-Ping Li, Shiang-Yi Lu, Yen-Jen Lin, Chih-Yen Chen

**Affiliations:** Department of Materials and Optoelectronic Science, National Sun Yat-Sen University, Kaohsiung 804, Taiwan; a0970919519@gmail.com (R.-P.L.); melenlu24@gmail.com (S.-Y.L.); hi60254ih@gmail.com (Y.-J.L.)

**Keywords:** Bi_2_Te_3_ nanosheets, environmental SEM, direct observations, growth mechanism, UV–Vis–NIR spectroscopy

## Abstract

As extraordinary topological insulators, 2D bismuth telluride (Bi_2_Te_3_) nanosheets have been synthesized and controlled with a few-layer structure by a facile and fast solvothermal process. The detail-oriented growth evolution of 2D Bi_2_Te_3_ in an ethylene glycol reducing solution is discovered and recorded for direct observation of the liquid–solid interactions through the use of environmental SEM. At the initial synthesis stage, Te nanowires are rapidly synthesized and observed in solution. In the next stage, Bi nanoclusters slowly adhere to the Te nanowires and react to form hierarchical Te-Bi_2_Te_3_ nanostructured materials. Additionally, the Te nanowires shorten in-plane in an orderly manner, while the Bi_2_Te_3_ nanosheets exhibit directional out-of-plane epitaxial growth. In the last procedure, Bi_2_Te_3_ nanosheets with a clear hexagonal appearance can be largely obtained. Experiments performed under these rigorous conditions require careful consideration of the temperature, time, and alkaline environment for each reaction process. In addition, the yield of a wider and thinner Bi_2_Te_3_ nanosheet is synthesized by manipulating the crystal growth with an optimal alkaline concentration, which is found through statistical analysis of the AFM results. In the UV–Vis–NIR spectroscopy results, the main peak in the spectrum tends to redshift, while the other peak in the ultraviolet range decreases during Bi_2_Te_3_ nanosheet synthesis, facilitating a rapid understanding of the trends in the morphological evolution of the Bi_2_Te_3_ materials in solution. By rationalizing the above observations, we are the first to report the success of environmental SEM, HAADF-STEM, and UV–Vis–NIR spectroscopy for confirming the Bi_2_Te_3_ nanosheet formation mechanism and the physical properties in the solvent media. This research promotes the future optimization of promising Bi_2_Te_3_ nanomaterials that can be used in the fabrication of thermoelectric and topological components.

## 1. Introduction

Two-dimensional layer-structured metal chalcogenides, such as bismuth telluride (Bi_2_Te_3_) thermoelectrics, have attracted considerable attention for their unique applications during the past decades because they are functional materials that can be broadly applied as topological insulators [1,2], 2D photodetectors [3,4,5] and electrodes [6], and energy harvesters [7,8,9,10], including the latest innovations from our recent research on nanogenerator achievements [10]. The crystal structure of Bi_2_Te_3_ thermoelectrics belongs to the rhombohedral (R$\bar{3}$m) space group, which is described by a hexagonal unit cell with lattice constants of a = 1.041 nm and c = 3.029 nm and a stacking sequence of Te(1)-Bi-Te(2)-Bi-Te(1) to form a quintuple layer. It should be noted that the bonding between two quintuple layers, Te(1)-Te(1), is bonded by weak van der Waals interactions, similar to two-dimensional materials, thus leading to the observation of numerous exciting physical phenomena and exotic properties [11,12].

Furthermore, ultrathin Bi_2_Te_3_ structured materials are classified as topological insulators, as they generally have unique topological properties because the band structure of the Bi_2_Te_3_ surface exhibits a single nondegenerate Dirac cone, resulting in high conductivity, high electron mobility, and a switchable electronic surface current with low energy loss, which is an attractive property in many industrial fields [6,13]. On the other hand, Bi_2_Te_3_ materials also exhibit outstanding thermoelectric properties, so a variety of synthesis processes have been developed, including a common chemical vapor deposition (CVD) process applied to the synthesis of Bi_2_Te_3_ nanosheets [14,15,16], and the other much more precise controlling method, namely, molecular beam epitaxial (MBE) growth, which has also been utilized for synthesizing high-quality Bi_2_Te_3_ ultrathin films [17,18]. Although the quality and thickness of Bi_2_Te_3_ thin films can be effectively controlled by using these processes, to overcome the extremely costly production of MBE and CVD synthesis processes, such as expensive vacuum facilities and sample size limitations in the systems, a fast and simple solution process has been explored to form inexpensive, large, and uniform Bi_2_Te_3_ ultrathin films [12,19,20].

However, the undesirable intermediate product and the different shapes of the Bi_2_Te_3_-Te hierarchical-structured nanomaterial composed of Bi_2_Te_3_ nanosheets and Te nanorods have also been obtained by controlling the rates of the competing reactions for the formation of Bi_2_Te_3_ and Te during the experimental process through two-step solvothermal methods [21]. This three-dimensional hierarchical Bi_2_Te_3_-Te heterostructure in which the chemical composition changes with the position will affect the qualities of the Bi_2_Te_3_ ultrathin film grown by chemical synthesis; thus, it is necessary to prevent the formation of the intermediate hierarchical Bi_2_Te_3_-Te heterojunction to effectively control the growth of the Bi_2_Te_3_ ultrathin film during synthesis. Bi_2_Te_3_-Te hierarchical-structured nanomaterials have also been fabricated by adding sucrose as a template and ethylene glycol (EG) as a reducing agent so that different sizes of heterostructured nanomaterials can be obtained [21].

The detail-oriented description of the formation of Bi_2_Te_3_ hexagonal nanosheets has seldom been reported thus far, but it is believed that determining the detailed growth mechanism of Bi_2_Te_3_ nanosheets is meaningful for further application. In this study, the detailed mechanism of forming Bi_2_Te_3_ nanosheets using a simple one-pot solvothermal method is discovered, and the role of different times, temperatures, and alkaline concentrations are also investigated thoroughly. We also examine and rationalize the shape/aspect ratio of the Bi_2_Te_3_ nanosheets under different growth conditions by using a statistical method with atomic force microscopy (AFM) and ultraviolet–visible–near–infrared (UV–Vis–NIR) spectroscopy. The experimental direct and indirect observation results via environmental scanning electron microscopy (SEM), high resolution transmission electron microscope (HRTEM), and high-angle annular dark-field scanning transmission electron microscopy (HAADF-STEM) mapping images show that Bi_2_Te_3_-Te hierarchical nanomaterials will be generated briefly in solution during the formation of Bi_2_Te_3_ nanosheets. These useful research results indicate that prospective materials for nanogenerators and thermoelectric applications can be further developed in the future.

## 2. Materials and Methods

### 2.1. Preparation and Synthesis of the Bi_2_Te_3_ Hexagonal Nanosheets

**Figure 1:**
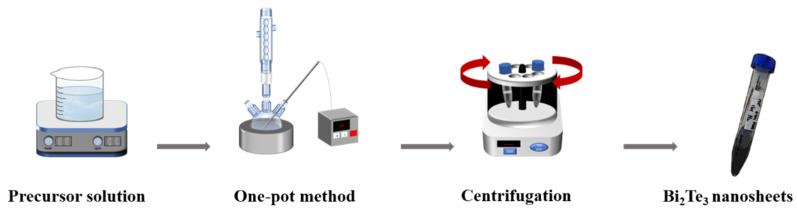
Schematic showing the growth process of the Bi_2_Te_3_ hexagonal nanosheets.

Bismuth nitrate pentahydrate (Bi(NO_3_)_3_·5H_2_O, 0.2 mmol, 99.99%, Acros, NJ, USA), sodium tellurite (Na_2_TeO_3_, 0.3 mmol, 99.5%, Alfa, Turin, Italy), polyvinyl pyrrolidone (2 mmol, Mw 40,000, Alfa) and a specific quantity of sodium hydroxide (NaOH, 97%, Showa, Tokyo, Japan) were dissolved in 10 mL of ethylene glycol (C_2_H_4_(OH)_2_, 99.8%, Sigma-Aldrich, St. Louis, MO, USA). To examine the role of chemical reactions in alkaline solutions, a variety of precise concentrations of NaOH with 0.2 M, 0.3 M, 0.4 M, and 0.5 M were prepared. The mixture required a heat reflux setup in a three-necked flask, which was heated and maintained at several controllable temperatures of 170 °C, 180 °C, and 190 °C. The synthesized sample was centrifuged at 8000 rpm for 8 min with a blended solvent of 5 mL of acetone (99.5%, Acros) and 10 mL of isopropanol (99%, Acros). The precipitates were dispersed and cleaned by an ultrasonicator three times. The final synthesized product was cleared by centrifugation and then dropped on a silicon substrate by using a spin-coating method to conduct further characterization. The experimental procedures are illustrated in Figure 1.

### 2.2. Material Characterization

For material characterization, X-ray diffraction (XRD, D8 Bruker) with a Cu-Kα radiation source (λ = 1.54 Å) and scanning electron microscopy (SEM, JEOL JSM-6330) equipped with an energy dispersive spectroscopy (silicon drift detector (SDD) EDS, AMETEK EDAX Genesis PV9840) detector at different takeoff angles (40°) were utilized to understand the crystal structure, morphology, and composition of the Bi_2_Te_3_ nanosheets. The scanning range was from 10° to 70°, and the scanning rate was 0.0125° per second in the XRD measurement. The software (MDI Jade 6.5) was used for XRD pattern analysis. The accelerating voltages were used in SEM between 10 and 15 kV. Next, the detailed high-resolution lattice structure and nanoscale elemental distribution of the Bi_2_Te_3_ nanosheets were further characterized by ultrahigh vacuum high-resolution scanning transmission electron microscopy (STEM, FEI Tecnai F20 G2) at an accelerating voltage of 200 keV with a point-to-point resolution of 0.14 nm and equipped with a high-angle annular dark field (HAADF) and EDS (SDD, OXFORD INCA x-stream) detector at different takeoff angles (15°). The EDS maps were formed using the X-ray lines: Bi-M and Te-L. For real-time liquid material analysis, environmental SEM testing techniques for observing material formation were investigated through a single-use sealable carrier with a microchannel inside (Aquarius Kit, FlowVIEW Tek). The experiment used nanofilm fabricated by microelectromechanical systems (MEMS) to create an atmospheric environment during SEM, thereby keeping the liquid samples in their original state. This sealable carrier is precise, facilitating the simultaneous direct observation of analytical results, such as the particle dispersity/aggregate, shape evolution, and composition distributions.

The synthesized sample was used to identify the thickness and size of the material with atomic force microscopy (AFM, Hitachi 5000). In this statistical investigation, ultrathin Bi_2_Te_3_ sheets were extracted in a dilute solvent by a spin-coating method, and thirty pieces of Bi_2_Te_3_ nanosheets were regularly recorded to determine the average correlation between the thickness and diameter by AFM. For the non-destructive chemical analysis technique, absorption spectrum analysis was performed with ultraviolet–visible–near-infrared (UV–Vis–NIR) spectroscopy (JASCO, V-770 EX) to measure the physical properties and morphological evolution of materials.

## 3. Results

### 3.1. Synthesis of the Bi_2_Te_3_ Nanosheets and Bi_2_Te_3_-Te Hierarchical Nanostructure under Different Temperature and Alkaline Conditions

**Figure 2:**
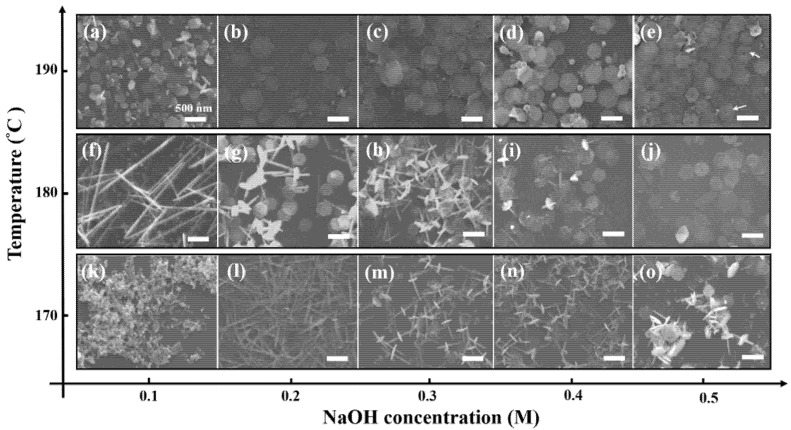
Investigating the correlation of concentration on the reaction temperature. The morphologies of the Bi_2_Te_3_ hexagonal nanosheets with different concentrations of NaOH reacting at (**a**–**e**) 190 °C, (**f**–**j**) 180 °C, and (**k**–**o**) 170 °C for 1 h. The concentrations of NaOH are arranged systematically (**a**,**f**,**k**) 0.1 M, (**b**,**g**,**l**) 0.2 M, (**c**,**h**,**m**) 0.3 M, (**d**,**i**,**n**) 0.4 M, and (**e**,**j**,**o**) 0.5 M. The scale bar is 500 nm. The ET detector is a secondary electron detector used in SEM.

To present the influence of temperature, the synthesis products with different alkaline concentrations at 170–190 °C for 1 h are displayed in Figure 2. The growth mechanism of the Bi_2_Te_3_ nanosheets at different temperatures (170–190 °C), indicates a higher reaction rate at both higher alkaline concentration and reaction temperature. Interestingly, the morphology of the Bi_2_Te_3_ nanosheets is completely different when reacted with different concentrations in the reaction temperature range of 170 °C to 190 °C, as shown in Figure 2.

With careful examination of the SEM images (Figure 2a–e), it can be found clearly that well synthesized hexagonal nanosheets are obtained in 0.1M, 0.2 M, 0.3 M, 0.4 M, and 0.5 M NaOH solution. Note that an increasing number of nuclei are formed in the higher alkaline concentration solution so that large quantities of Bi_2_Te_3_ nanosheets are consequently produced. Interestingly, some slight nanopores (10 nm in diameter) are observed in the middle of the Bi_2_Te_3_ nanosheets that reacted with 0.5 M NaOH at 190 °C for 1 h. The results are indicated by the white arrows shown in Figure 2e. Previous literature reported that nanopores were found in the middle of Bi_2_Te_3_ nanosheets below a reaction temperature of 190 °C using the solvothermal method; however, no nanopores appeared in the Bi_2_Te_3_ nanosheets above a relatively high reaction temperature of 200 °C [12]. The pores provide important evidence for growth from nanowires to nanosheets. In 0.5 M NaOH solution, Te atoms diffused from the nanowires to form Bi_2_Te_3_ nanosheets, which were intercepted because the reaction time of 1h was not sufficient. Once the reaction time is extended to 3h, the Bi and Te atoms will diffuse uniformly to the entire hexagonal sheet until the final hole is repaired, as shown in Figure S1 in the Supplementary File.

In Figure 2f, there are many fairly tiny and smooth nanowires with diameters of 80–100 nm and no complete hexagonal Bi_2_Te_3_ nanosheets. However, many Bi_2_Te_3_-Te hierarchical nanostructures, which exhibit a lot of Bi_2_Te_3_ nanosheets perpendicular to the Te nanowires, are observed and recorded in Figure 2g–i and Figure 2l–o, representing the batch experiments of 0.2 M, 0.3 M, and 0.4 M NaOH reacted at 170 °C and 180 °C for 1 h. Notably, no Bi_2_Te_3_ nanosheets and Te nanowires could be observed in Figure 2k.

**Figure 3:**
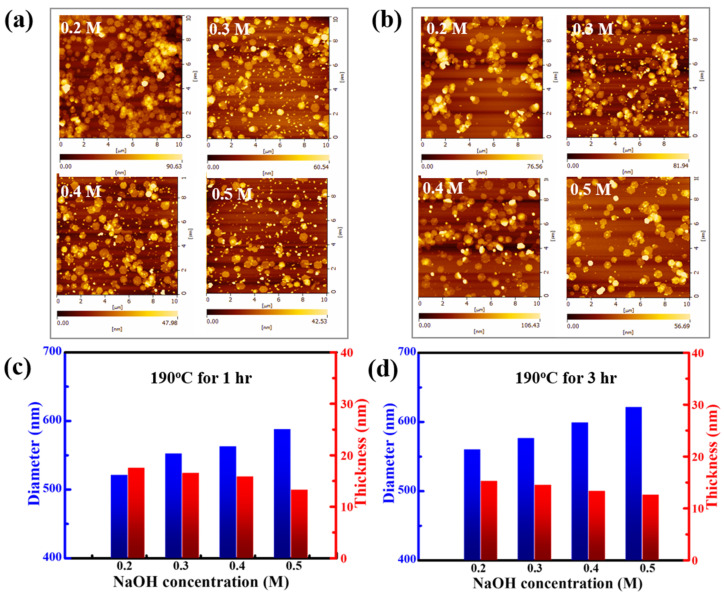
AFM images of the Bi_2_Te_3_ hexagonal nanosheets synthesized at 190 °C at various concentrations of 0.2 M, 0.3 M, 0.4 M, and 0.5 M NaOH for (**a**) 1 h and (**b**) 3 h, respectively. Statistical histogram of the mean size and thickness distribution of Bi_2_Te_3_ nanosheets at different NaOH concentrations for (**c**) 1 h and (**d**) 3 h at 190 °C, respectively.

AFM images show the average distribution of the thickness and size of thirty pieces of Bi_2_Te_3_ nanosheets synthesized at 190 °C for different reaction times, namely, 1 h (Figure 3a) and 3 h (Figure 3b). The completed data of the statistical mean size and thickness distribution of Bi_2_Te_3_ nanosheets are summarized in Figures S2 and S3 in the Supplementary File. When the reaction time is increased, the material tends to grow laterally. In Figure 3c,d, the statistical results show that when the average diameter of Bi_2_Te_3_ nanosheets is significantly increased, the average thickness will be thinner. These measurement results are very important for future practical applications. A previous report stated that some metal chalcogenide nanosheets can be studied with AFM in terms of their internal size and thickness. It indicates a larger size-to-thickness ratio during synthesis; thus, the formation of morphology is dominated by nanosheets [22].

According to the statistical results of the AFM measurements, even though the reaction time is increased from 1 h to 3 h, the average thickness of an individual Bi_2_Te_3_ nanosheet is approximately 15 nm. However, the average thickness of the Bi_2_Te_3_ nanosheets tends to become thinner with increasing concentrations of NaOH. The overall thickness of the Bi_2_Te_3_ nanosheet tends to become thicker while the reaction time is increased from 1 h to 3 h, and the average of the Bi_2_Te_3_ nanosheets decreases from 13 nm to 16 nm. In terms of size, the diameter distribution of the Bi_2_Te_3_ nanosheets is approximately 520–620 nm. It can be observed that the average diameter of the Bi_2_Te_3_ nanosheets becomes larger with a higher NaOH concentration, and the average size of the Bi_2_Te_3_ nanosheets that are produced after reacting at 190 °C for 1 h and 3 h increases from 520 nm to 580 nm and from 560 nm to 620 nm, respectively. The AFM results show that the overall size of the Bi_2_Te_3_ nanosheets tends to increase as the reaction time is extended from 1 h to 3 h.

### 3.2. Suggested Mechanism and UV–Vis–NIR Spectroscopy Results of Bi_2_Te_3_-Te Hierarchical Nanostructure and Bi_2_Te_3_ Nanosheets

**Figure 4:**
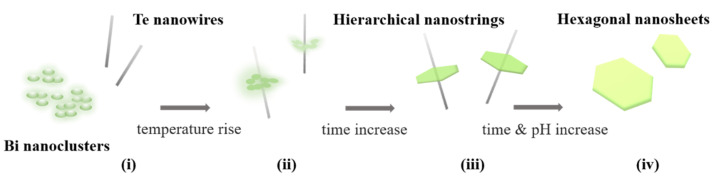
Suggested self-assembly mechanism of the Bi_2_Te_3_ hierarchical nanostructure. (**i**) Te nanowire/Bi nanoclusters, (**ii**) Te nanowire/Bi_2_Te_3_ platelet composites, (**iii**) Bi_2_Te_3_ hierarchical structure, and (**iv**) Bi_2_Te_3_ nanosheets.

According to the results of SEM in Figure 2, a perspective self-assembly mechanism of the Bi_2_Te_3_ hierarchical nanostructure is illustrated in Figure 4. In this study, Te nanowires are observed to first synthesize in the solution before the bismuth nitrate pentahydrate solution is injected to form the hierarchical Bi_2_Te_3_-Te heterogeneous structure. Furthermore, a simultaneous synthesis process presents the transformation from pure chalcogen and metal particles to form metal chalcogenides. This finding is similar to the previously investigated nonstoichiometric nucleation and growth mechanism of two-dimensional nanosheets of layer-structured metal chalcogenides [23,24].

**Figure 5:**
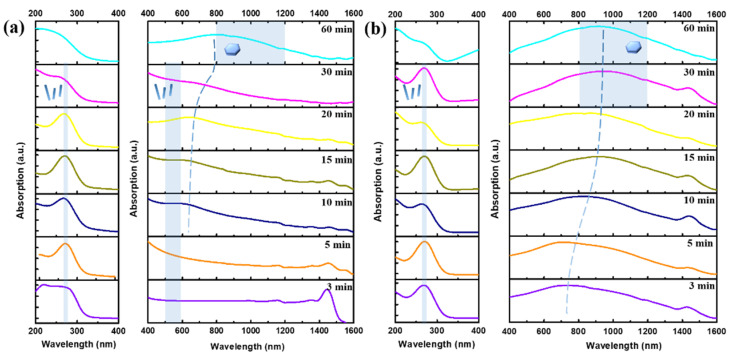
UV–Vis–NIR absorption measurements were obtained over a reaction time of 3, 5, 10, 15, 20, 30, and 60 min at (**a**) 170 °C and (**b**) 190 °C in 0.4 M NaOH.

To study the growth evolution of Bi_2_Te_3_ nanosheets and the correlation of their optical properties, UV–Vis–NIR absorption spectroscopy was subsequently adopted with samples synthesized in 0.4 M NaOH reacting at 170 °C and 190 °C for seven different reaction periods of 3, 5, 10, 15, 20, 30, and 60 min, as shown in Figure 5a,b, respectively.

In the SEM results for different reaction time (Figure S4 in the Supplementary File), it can be seen that Te nanowires appear at 170 °C after 10 min, as shown in Figure S4d–f; additionally, few Bi_2_Te_3_ nanosheets are formed and attached to the Te nanowires. When the reaction time is extended to 30 min, a high density and yield of Bi_2_Te_3_ hierarchical heterostructure are obtained. From the related literature on the absorption spectrum of Te nanowires, a significant absorption peak exists at 280 nm and 630 nm [25]. The reason is the electron transitions from the valence band (p-lone pair VB3) to the conduction band (p-antibonding CB1) [25]. Thus, it is possible to determine that the absorption peaks at 280 nm and 630 nm in Figure 5a are caused by the Te nanowires. The reason for the occurrence of a red shifting absorption peak recorded from 20 min to 60 min during the growth process is that the Te atoms and Bi atoms gradually undergo a chemical reaction to form Bi_2_Te_3_ nanosheets as shown in Figure 5a.

In Figure S5a–d, it can be seen that the morphology evolution from Bi_2_Te_3_-Te hierarchical nanostructure to predominantly Bi_2_Te_3_ nanosheets appeared after 3 min to 15 min at 190 °C, which leads to the occurrence of a red shifting absorption peak during the initial growth process. Furthermore, it can be observed that only the Bi_2_Te_3_ nanosheets are present after the reaction lasts for 20 min, and no other types of nanostructured materials are presented in Figure S5e,f, which shows a stable broad absorption in the range of 800~1000 nm as shown in Figure 5b. It is known from the literature that the characteristic absorption spectrum of Bi_2_Te_3_ nanosheets is a broad absorption peak from visible light to infrared light [20,25,26].

As seen from the measurement results in Figure 5b, the absorption peak at a wavelength of 650 nm in the three-minute experiment appears to be redshifted to the infrared light range (800~1000 nm). Some studies reported that the incident-free electrons were absorbed on the surface of Bi_2_Te_3_ nanosheets and interacted to generate plasmon resonance, thus causing a large amount of light absorption in the infrared light range [20,26]. In addition, the absorption peak broadens for two reasons. First, the surface of Bi_2_Te_3_ nanosheets has three hexagonal surface plasmon modes. According to the electron energy loss spectroscopy (EELS) experimental results in the literature, the energy is located at 1.6, 2.1, and 3.1 eV, which relates to the size distribution of Bi_2_Te_3_ nanosheets [20,26]. Herein, it can be confirmed that the growth mechanisms at both 170 °C and 190 °C are similar, but the only difference is the reaction rate.

### 3.3. TEM Characterization of the Predicted Growth Mechanism and Chemical Reaction Formula of the Bi_2_Te_3_ Nanosheets and Bi_2_Te_3_-Te Hierarchical Nanostructure

**Figure 6:**
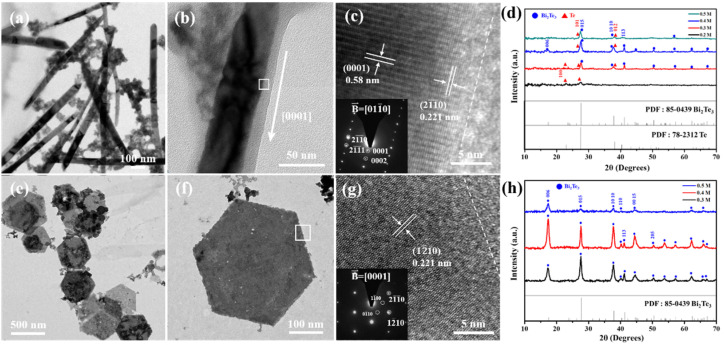
TEM was used to investigate the role of Te nanowires and Bi_2_Te_3_ hexagonal nanosheets. Bright-field images of all Te nanowires and Bi_2_Te_3_ nanosheets. The Te nanowires were synthesized in 0.2 M NaOH solvent at 170 °C for 1 h. (**a**) Lower magnification TEM bright-field image of pure Te nanowires with diameters of 80–100 nm and (**b**) high magnification indicating the growth direction of the nanowire along with the [0001] zone. (**c**) HRTEM image and the corresponding SAED patterns of the white square in (**b**). (**d**) XRD patterns of the Te-Bi_2_Te_3_ nanocomposites reacted with various NaOH concentrations at 170 °C. The Bi_2_Te_3_ nanosheets were synthesized in 0.3 M NaOH solvent at 190 °C for 1 h. (**e**) Lower magnification TEM bright-field image of Bi_2_Te_3_ nanosheets with diameters of 500 nm and (**f**) high magnification image of an individual Bi_2_Te_3_ nanosheet existing on the {1$\bar{1}$00} plane. (**g**) HRTEM and corresponding SAED pattern images are shown in the white square in (**f**). (**h**) XRD patterns of the hexagonal Bi_2_Te_3_ nanosheets reacted with various NaOH concentrations at 190 °C for 1 h.

The detailed TEM characterization is investigated in Figure 6, in which Figure 6a shows the bright-field image of the Te nanowires synthesized at 170 °C with 0.2 M NaOH for 1 h, and the representative XRD patterns of the Te nanowires belong to a hexagonal structure with space group P3_1_21, as shown in Figure 6d. It is also observed that many Bi nanoclusters are entangled with the nanowires in Figure 6a. In Figure 6b,c, the higher magnification of the bright-field image and its corresponding high-resolution TEM image present the nanowire that grew in the [0001] direction. Figure 6c also shows a length of 0.221 nm of the corresponding (2$\bar{1}\bar{1}$0) d-spacing, and the SAED patterns of the Te nanowire are recorded along with the [01$\bar{1}$0] zone axis.

On the other hand, the many pieces of Bi_2_Te_3_ nanosheets from reaction with 0.3 M NaOH at 190 °C for 1 h are presented in Figure 6e, and the morphology of an individual Bi_2_Te_3_ nanosheet presents a good hexagonal shape, as shown in Figure 6f. It should be noted that the edges of the hexagonal nanosheets are determined to be the {1$\bar{1}$00} planes. According to the literature [27], the surface energy of {1$\bar{1}$00} is lower than that of {$\bar{2}$110}, indicating that the formation of the {1$\bar{1}$00} edge is more energetically favorable. The corresponding high-resolution TEM image of the white square in Figure 6f is presented in Figure 6g, and the insert shows the SAED patterns characterizing the Bi_2_Te_3_ rhombohedral structure with a [0001] zone axis. The representative XRD analysis of the Bi_2_Te_3_ nanosheets also shows a rhombohedral structure with space group R$\bar{3}$m, as shown in Figure 6h.

**Figure 7:**
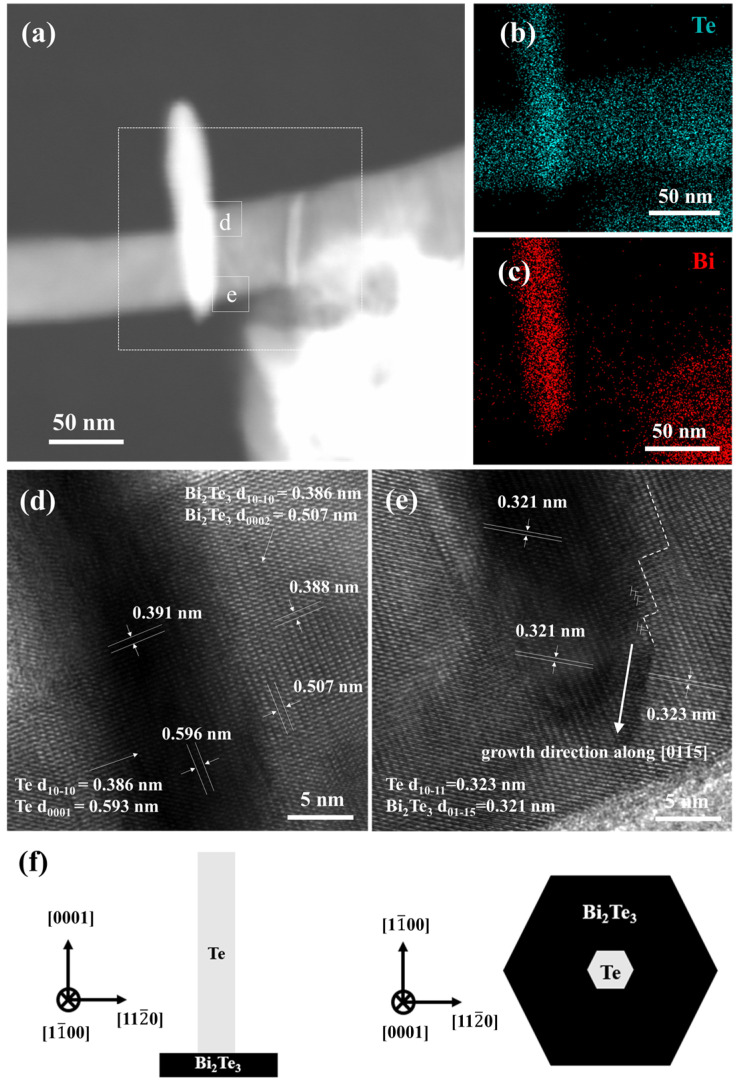
(**a**) HAADF-STEM image of the Bi_2_Te_3_-Te hierarchical heterostructure synthesized at 170 °C for 1 h in 0.3 M NaOH. EDS elemental mapping results of (**b**) Te and (**c**) Bi. (**d**,**e**) HRTEM images of the squares representing Regions d and e in (**a**), respectively. (**f**) Schematic diagram showing the crystal orientation of the Bi_2_Te_3_-Te hierarchical heterostructure.

To further understand the predicted growth mechanism of the Bi_2_Te_3_ nanosheets, a Bi_2_Te_3_-Te hierarchical nanostructure was observed directly under TEM, as shown in Figure 7. In Figure 7a, the appearance of the Bi_2_Te_3_-Te heterostructure is observed under the above parameters. The EDS analysis for elemental mapping in HAADF-STEM images (Figure 7b,c) can further confirm that the composition of the nanowire is ultra-pure Te, and the Bi/Te ratio of the nanosheets (white square) is close to the chemical dose ratio of Bi_2_Te_3_. The early stage of the Bi_2_Te_3_ nanosheets undergoes oriented attachment, while the Te atoms continue to diffuse to the surface, reacting with the Bi precursor and shortening the length of the Te nanowires.

Furthermore, the HRTEM images of the white square representing Region d in Figure 7d show that the interplanar distances are calculated to be approximately 0.391 nm and 0.596 nm, which are nearly consistent with the literature values of the Te (10$\bar{1}0$) and (0001) planes, respectively [28]. The interplanar distances are calculated to be approximately 0.388 nm and 0.507 nm, which are also consistent with the reported values of the Bi_2_Te_3_ (10$\bar{1}0$) and (0002) planes, respectively [28]. Additionally, the lattice relationship in the HRTEM images of the white square representing Region e, as shown in Figure 7e, is investigated, and the interplanar distances are calculated to be approximately 0.323 nm and 0.321 nm, which are nearly consistent with those of the Te (10$\bar{1}1$) and Bi_2_Te_3_ (01$\bar{1}5$) planes, respectively. It is also worth noting that a very high density of dislocations accumulates at the Bi_2_Te_3_-Te interface; therefore, the growth direction of Bi_2_Te_3_ nanosheets advances in a zigzag shape along with the [01$\bar{1}5$] direction. The bent edge of the Bi_2_Te_3_ nanosheets becomes straight to reduce the surface energy, resulting in the formation of the {1$\bar{1}$00} edge. In addition, the crystal orientation relationship between the hierarchical Bi_2_Te_3_-Te heterojunction atoms is illustrated in Figure 7f. It can be understood that the (0001) Te *||* (0002) Bi_2_Te_3_ and [11$\bar{2}0$] Te *||* [11$\bar{2}0$] Bi_2_Te_3_ [28]. Finally, a clearly outlined Bi_2_Te_3_ nanosheet form is successfully obtained at a late stage of the process.

## 4. Conclusions

In summary, the detailed growth mechanism of Bi_2_Te_3_ by using a simple one-pot solvothermal method is thoroughly understood. Several steps were sequentially observed in the solution. Te ion was first reduced by the hydroxide group to form Te nanowires by self-assembly. Second, Bi precursors adhered to the Te nanowires and reacted into hierarchical Te-Bi_2_Te_3_ nanostructured materials. Third, the Te atoms continuously diffused from the Te nanowire to the Bi-Te interface, providing the Te required for nanosheet growth and shortening the Te nanowire. Finally, the perfect morphological features of the Bi_2_Te_3_ nanosheets were successfully formed.

In addition, a higher alkaline concentration and lower reaction temperature resulted in wider and thinner Bi_2_Te_3_ nanosheets, as shown in the AFM statistical analysis. With increasing base concentration, more hydroxide ions act as reducing agents, leading to an increase in the yield of Bi_2_Te_3_ nanosheets with perfect morphology. As for increasing reaction temperature or time, it increases the motion of ions in solution in in-plane and out-of-plane directions and the diffusion of Te atoms into the Te nanowires, resulting in a little thicker Bi_2_Te_3_ nanosheets. The higher NaOH concentration simultaneously leads to faster growth along the out-of-plane direction, resulting in a larger size and thinner Bi_2_Te_3_ nanosheets.

On the other hand, the use of UV–Vis–NIR spectroscopy is helpful to rapidly understand the morphological evolution of Bi_2_Te_3_ nanosheets in solution. The main broad peak on the spectrum tended to be red-shifted, while the other sharp peak in the ultraviolet range weakened during the synthesis of Bi_2_Te_3_ nanosheets. By rationalizing the above series of observations, we report for the first time that environmental SEM, TEM, and UV–Vis–NIR spectroscopy successfully confirm the growth mechanism of Bi_2_Te_3_ nanosheet through the liquid–solid formation. This research constructively provides an optimized process for Bi_2_Te_3_ nanosheets, which shows promising applications as thermoelectrics, topological insulators, and nanogenerators in future micro and nanoelectronic devices.

## Data Availability

Data presented in this article are available at request from the corresponding author.

## Supplementary Materials

The following supporting information can be downloaded at: https://www.mdpi.com/article/10.3390/nano12132236/s1, Figure S1. SEM images of the hexagonal Bi_2_Te_3_ nanosheets synthesized in (a) 0.2 M, (b) 0.3 M, (c) 0.4 M, and (d) 0.5 M NaOH at 190 °C for 1 h. The hexagonal Bi_2_Te_3_ nanosheets synthesized in (e) 0.2 M, (f) 0.3 M, (g) 0.4 M, and (h) 0.5 M NaOH at 190 °C for 3 h. Figure S2: The histograms of size distributions of Bi_2_Te_3_ nanosheets from Figure 3a,b. Figure S3: The histograms of thickness distributions of Bi_2_Te_3_ nanosheets from Figure 3a,b. Figure S4. SEM images of the changes in the morphology of the hexagonal Bi_2_Te_3_ nanosheets synthesized in 0.4 M NaOH at 170 °C for different reaction times: (a) 3 min, (b) 5 min, (c) 10 min, (d) 15 min, (e) 20 min, and (f) 30 min. The scale bar in the figure is 1 micrometer, and the scale bar in the inset figure is 400 nanometers. Figure S5. SEM images of the changes in the morphology of the hexagonal Bi_2_Te_3_ nanosheets synthesized in 0.4 M NaOH at 190 °C for different reaction times: (a) 3 min, (b) 5 min, (c) 10 min, (d) 15 min, (e) 20 min, and (f) 30 min. The scale bar in the figure is 1 micrometer, and the scale bar in the inset figure is 400 nanometers.
