# Supplementary material for: Direct Observation of the Epitaxial Growth of Bismuth Telluride Topological Insulators from One-Dimensional Heterostructured Nanowires
Source: Nanomaterials (Basel). 2022 Jun 29;12(13):2236. doi: 10.3390/nano12132236 (PMC9268475; doi:10.3390/nano12132236)
Supplement: Supplementary file 1 [file nanomaterials-12-02236-s001.zip › nanomaterials-1757546-supplementary.pdf]

nanomaterials

Supplementary File

Direct Observation of the Epitaxial Growth of Bismuth Telluride Topological Insulators from One-Dimensional Heterostructured Nanowires

Rei-Ping Li †, Shiang-Yi Lu †, Yen-Jen Lin and Chih-Yen Chen *

Department of Materials and Optoelectronic Science, National Sun Yat-Sen University, Kaohsiung 804, Taiwan; a0970919519@gmail.com (R.-P.L.); melenlu24@gmail.com (S.-Y.L.); hi60254ih@gmail.com (Y.-J.L.)

* Correspondence: cychen@mail.nsysu.edu.tw

† These authors contributed equally to this work.

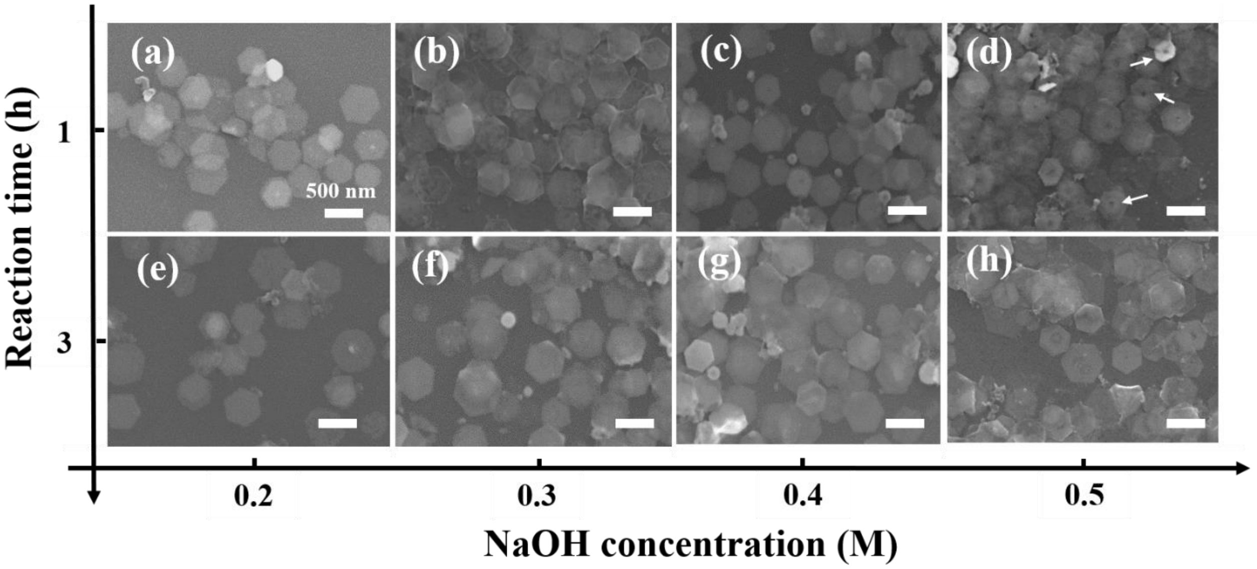

Figure S1. SEM images of the hexagonal Bi_2Te_3 nanosheets synthesized in (**a**) 0.2 M, (**b**) 0.3 M, (**c**) 0.4 M, and (**d**) 0.5 M NaOH at 190°C for 1h. The hexagonal Bi_2Te_3 nanosheets synthesized in (**e**) 0.2 M, (**f**) 0.3 M, (**g**) 0.4 M, and (**h**) 0.5 M NaOH at 190°C for 3h.

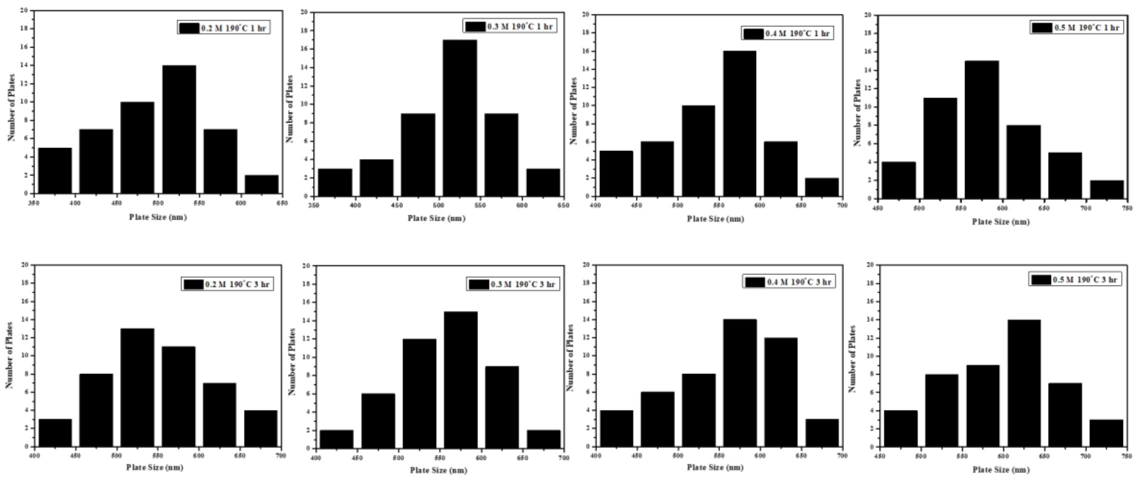

Figure S2. The histograms of size distributions of Bi_2Te_3 nanosheets from Figures 3a and 3b.

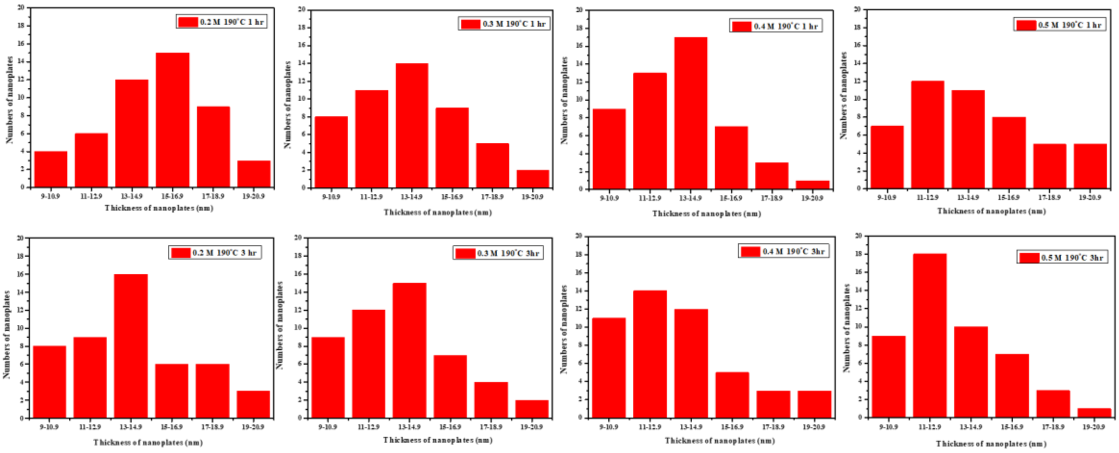

Figure S3. The histograms of thickness distributions of Bi_2Te_3 nanosheets from Figures 3a and 3b.

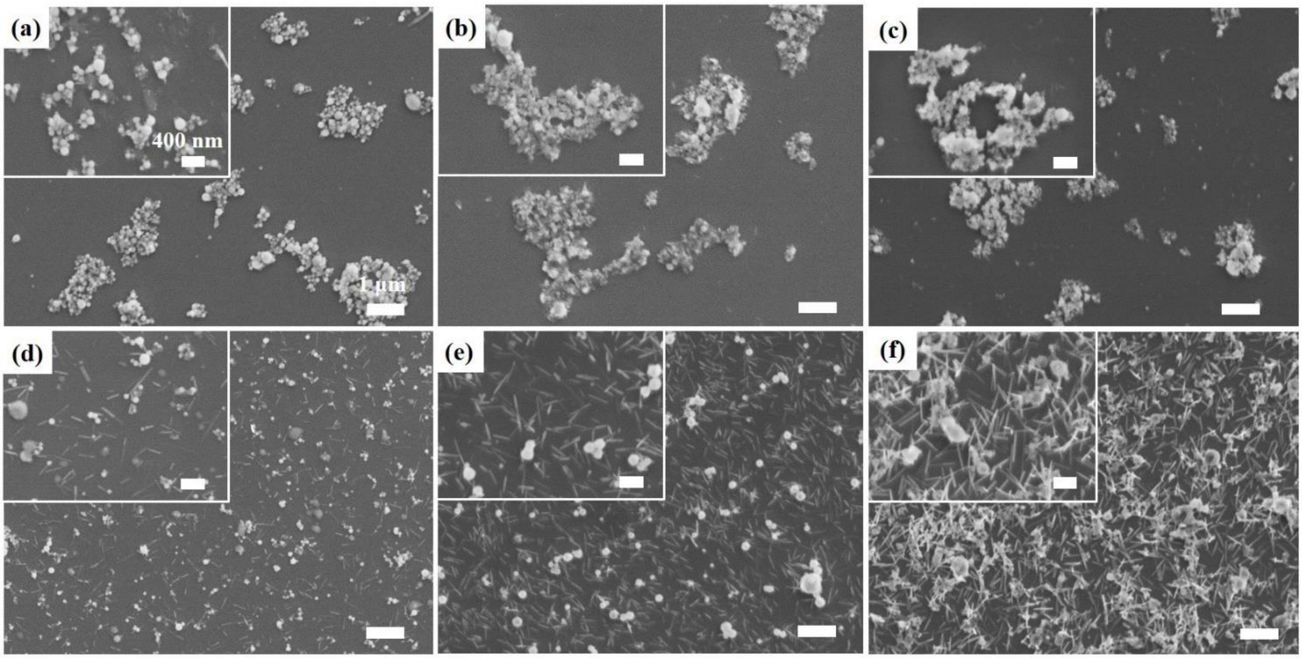

Figure S4. SEM images of the changes in morphology of the hexagonal Bi_2Te_3 nanosheets synthesized in 0.4 M NaOH at 170°C for different reaction times: (**a**) 3 min, (**b**) 5 min, (**c**) 10 min, (**d**) 15 min, (**e**) 20 min, and (**f**) 30 min. The scale bar in the figure is 1 micrometer, and the scale bar in the inset figure is 400 nanometers.

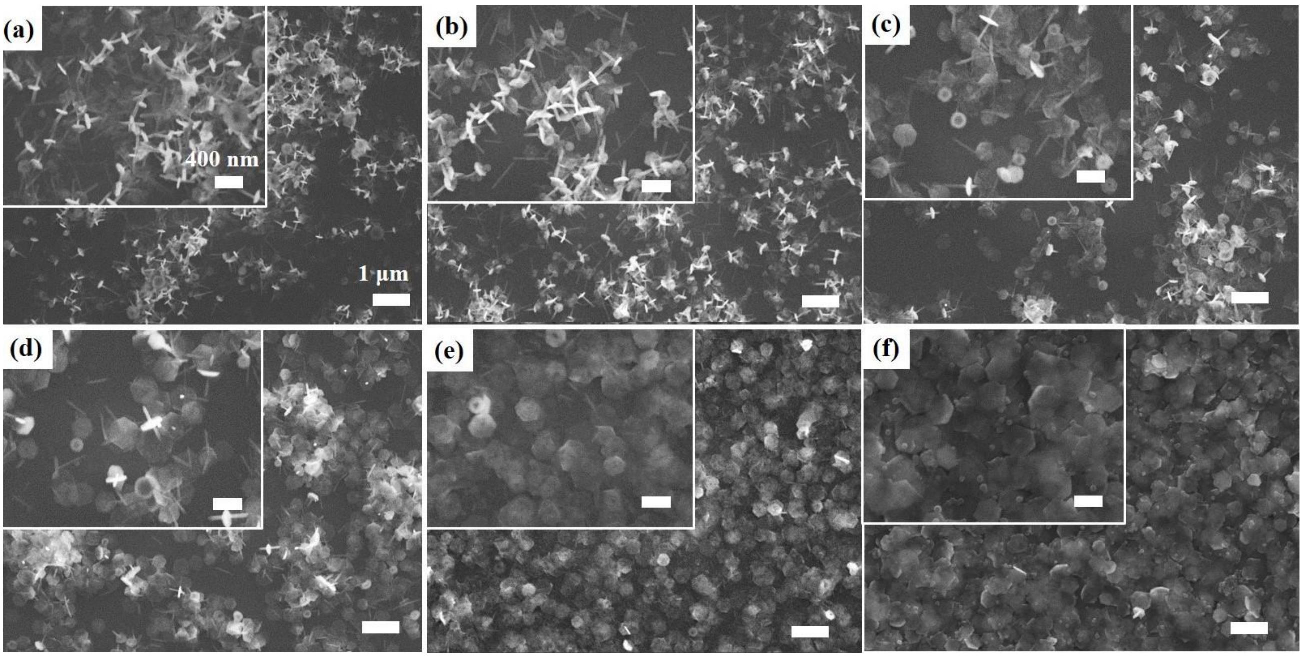

Figure S5. SEM images of the changes in morphology of the hexagonal Bi_2Te_3 nanosheets synthesized in 0.4 M NaOH at 190°C for different reaction times: (**a**) 3 min, (**b**) 5 min, (**c**) 10 min, (**d**) 15 min, (**e**) 20 min, and (**f**) 30 min. The scale bar in the figure is 1 micrometer, and the scale bar in the inset figure is 400 nanometers.
